# Association between APOBEC3H-Mediated Demethylation and Immune Landscape in Head and Neck Squamous Carcinoma

**DOI:** 10.1155/2020/4612375

**Published:** 2020-07-24

**Authors:** Qin Liu, Yue-wen Luo, Ruo-yan Cao, Xue Pan, Xi-juan Chen, Si-yuan Zhang, Wei-lin Zhang, Jia-ying Zhou, Bin Cheng, Xian-yue Ren

**Affiliations:** ^1^Hospital of Stomatology, Guanghua School of Stomatology, Sun Yat-sen University, Guangdong Provincial Key Laboratory of Stomatology, Guangzhou, Guangdong, China; ^2^School of Medicine, Sun Yat-sen University, Shenzhen, China

## Abstract

Immunotherapy has been demonstrated as a promising strategy in controlling head and neck squamous cell carcinoma (HNSC). The AID/APOBEC family is well characterized as DNA mutator and considered to play critical roles in immune responses in HNSC. However, the expression pattern and deamination-dependent demethylation roles of AID/APOBECs in HNSC are unclear. In this study, the RNA-seq and DNA methylation profiles of HNSC from TCGA database and cell-based experiments were applied to analyze the relationships between AID/APOBEC expression levels, patients' clinical outcomes, methylation alterations, and immune responses. Here, we found that APOBEC3H was abnormally upregulated in HNSC patients. HPV+ patients tended to have higher APOBEC3H levels than HPV- patients. Remarkably, patients with high APOBEC3H levels showed a favorable overall survival. Furthermore, tumors with high APOBEC3H levels exhibited a genome-wide DNA hypomethylation pattern. APOBEC3H was identified to demethylate and upregulate CXCL10 and improve CD8+ T cell tumor infiltration in the tumor microenvironment. Collectively, APOBEC3H plays critical roles in CD8+ T cell immune infiltration and activation in HNSC, which may be a potential biomarker for oncoimmunotherapy in HNSC.

## 1. Introduction

Head and neck cancer (HNC), which encompasses a group of malignancies arising from the upper digestive tract, salivary glands, and thyroid, is the sixth most common cancer in the world. Over 830,000 individuals are diagnosed and 430,000 individuals are dead with HNSC per year [1]. The strongest risk factors for HNSC are cigarette smoking and alcohol drinking [2–4]. Human papillomavirus (HPV) infection is associated with the increasing incidence of HNSC [5]. Although surgery and/or chemoradiotherapy have been routinely applied in the clinical management of HNSC patients, the 5-year overall survival rate is still below 60% [6]. HNSC is reported to be immunologically “hot” tumor with high immune cell infiltration, indicating that immune therapy may provide a promising strategy for HNSC patient treatment [7]. However, there are only 15-20% of HNSC patients with a moderate-high mutational burden who respond to PD-1/PD-L1 checkpoint blockade (ICB) immunotherapy [8, 9]. Thus, it is essential to explore the potential molecules that influence the HNSC immune microenvironment, which may provide biomarkers and therapeutic targets for HNSC patients.

The activation-induced cytidine deaminase/apolipoprotein B mRNA editing catalytic polypeptide-like (AID/APOBEC) family, which shares the homologous structural and catalytic backbone of zinc-dependent deaminases, is well established for its cytidine deaminase activity in RNA or single-strand DNA (ssDNA) and is essential for genome modulation, antibody diversity, and retroviral restriction [10–12]. In humans, there are 11 family members that have been identified, including AID, APOBEC1, APOBEC2, APOBEC3A-H, and APOBEC4. In a large number of cell-based experiments and biochemical assays, the AID, APOBEC1, and APOBEC3 proteins have been identified to deaminate cytosine to uracil (C-to-U) on RNA and DNA. DNA cytosine deamination is established as the hallmark activity of APOBECs due to the catalyzed deamination of HIV-1 cDNA replication intermediates during reverse transcription. APOBEC deaminating cytosine to uracil (C-to-U) in ssDNA is considered to be the most common event [13, 14]. DNA repair intermediates, such as DNA breaks and abasic sites, can also lead to cytosine to guanine (C-to-G) transversion and other mutational outcomes [15]. Thus, all the AID/APOBEC family members except for APOBEC2 and APOBEC4 were identified as DNA mutators [16]. In the last decades, a novel role of genomic cytosine demethylation activity has been reported in several AID/APOBEC family members [16]. Despite the role of genomic cytosine demethylation by AID remains controversial, APOBEC3A, APOBEC3B, and APOBEC3H have been demonstrated to have actual activity in cytosine demethylation in vitro. AID/APOBEC-mediated demethylation might be induced via deaminating 5mC and generating a T-G mismatch, which subsequently repaired by the base excision repair (BER) enzyme-thymidine DNA glycosylase [17]. However, the role of deamination-dependent demethylation of AID/APOBECs is poorly understood and remains to be further elucidated.

AID/APOBEC enzymes play critical roles in several cellular biological processes and pathological progression. They could initiate viral genome mutations, antibody somatic hypermutation, or class switching through targeting host genome immunoglobulin loci, which are essential for both adaptive and innate immune responses such as antiviral, B cell affinity maturation, or B cell class switch recombination [11, 12]. AID/APOBEC-mediated mutagenesis has been found to fuel for cancer heterogeneity and evolution. Although all the AID/APOBEC members mutate C to U, individual preferences are different. For example, AID prefers to deaminate the hotspot WRC (W corresponds to A/T, R means A/G, and C is the mutated cytosine), APOBEC3A and APOBEC3B exhibit preference for TC, while APOBEC3G prefers to mutate CCC. On the other hand, APOBEC3C, APOBEC3F, and APOBEC3H preferentially deaminate TTC [18, 19]. These patterns are reported to be related to the biological mechanisms underlying carcinogenesis [20, 21]. Thus, the abnormal expression pattern of AID/APOBECs has become a rising concern in human cancers. APOBEC3B is found to be upregulated in various cancer types and acts as a leading candidate for cancer mutator, such as breast cancer, non-small-cell lung cancer, and serous ovarian carcinoma [20, 22–24]. Another possible mutator-APOBEC3A can activate the DNA damage response, cause cell cycle arrest, and lead to cell death [25]. In HNSC, APOBEC mutagenesis was also found to trigger immune cell infiltration and activation, especially in HPV-mediated HNSC [26]. However, besides the mutagenesis activity, the biological functions and mechanisms of AID/APOBEC deaminases in HNSC progression remain largely unclear and need to be further elucidated.

In this study, the expression patterns, distinct prognostic values, methylation regulation activity, potential biological functions, and mechanisms of AID/APOBEC deaminases in HNSC were explored. We identified that APOBEC3H was obviously upregulated in HNSC, especially in those with positive HPV infection, and was a prognosis predictor. High APOBEC3H level was linked to the genome-wide aberrant hypomethylation in HNSC. APOBEC3H-mediated CXCL10 demethylation and expression might be responsible for CD8+ T cell infiltration and activation in HNSC, demonstrating a potential role of APOBEC3H in HNSC immunotherapy.

## 2. Materials and Methods

### 2.1. Clinical Cohorts

The HNSC dataset from the TCGA database (The Cancer Genome Atlas, Provisional) including 530 patients and 74 normal people was applied for this study. DNA methylation profiling based on Illumina Human Methylation 450 BeadChip, RNA-seq data based on IlluminaHiSeq RNASeq, and respective clinical information were obtained from the TCGA data portal.

### 2.2. Bioinformatics Analysis

Students' *t*-test was used to compare the expression levels of AID/APOBECs in HNSC and normal epithelial tissues. Fold change > 1.2 and *p* value < 0.05 were considered significantly differently expressed. The UALCAN dataset (http://ualcan.path.uab.edu/) was applied to analyze the expression levels of AID/APOBECs in HPV+ and HPV- samples [27]. R language was used for the Kaplan-Meier curves with log-rank test analysis. The best cutoff values for high and low AID/APOBEC expressions were selected from the receiver operating characteristic (ROC) curve. APOBEC1 and APOBEC4 were excluded from this study for more than 50% patients' mRNA data were not available in TCGA dataset.

The cBioportal (http://www.cbioportal.org) was applied to analyze the coexpression pattern of CXCL10 and APOBEC3H. TIMER dataset (https://cistrome.shinyapps.io/timer/) [28] was employed to perform enrichment analysis of genes that were correlated with APOBEC3H. The LinkInterpreter module was used for analyzing the genes which were positively correlated with APOBEC3H. The LinkFinder module was applied to analyze the correlation between APOBEC3H mRNA levels and CXCL10 methylation levels. The Kaplan-Meier survival module was performed to analyze the predictive value of CD8+ T cell infiltration on patients' overall survival. Furthermore, the immune cell infiltration data downloaded from the CIBERSORT (https://cibersort.stanford.edu/) [29, 30] was used to confirm the correlation between APOBEC3H or CXCL10 gene expression and CD8+ T cell infiltrations, as well as the prognostic value of the CD8+ T cell infiltrations in HNSC.

### 2.3. Methylation Analysis

To identify the potential role of APOBEC3H in HNSC, the patients were classified into three groups based on APOBEC3H mRNA levels: the top 30% were divided into high expression group, while those with bottom 30% expression levels were divided into low expression group. The differentially methylated CpG genes (DMGs) were identified by the ChAMP package in R language: delta beta > 0.15, adjusted *p* value < 0.05. The differentially expressed genes (DEGs) were identified by DEseq2 package in R language: log2FC > 1, adjusted *p* value <0.05. The Spearman correlation analysis was employed to seek the genes that were associated with APOBEC3H. Absolute correlation coefficient and *p* value < 0.05 were used as cutoff criteria.

Gene Ontology (GO) enrichment analysis and Kyoto Encyclopedia of Genes and Genomes (KEGG) pathways were performed using the clusterProfiler package and a *q* value < 0.05 as considered to represent statistical significance.

### 2.4. Cell Culture

The primary normal oral epithelium cell line HOK, the oral epithelium dysplasia cell line DOK, and HNSC cell lines (UM1, SCC1, HSC3, HSC6, CAL27, CAL33, and HN6) were maintained in our laboratory and had been authorized before use. SCC1, HSC3, HSC6, CAL27, and CAL33 were cultured in Dulbecco's modified Eagle medium (Gibco) supplemented with 10% fetal bovine serum (FBS, Gibco). UM1 and HN6 were grown in DMEM/F-12 (Gibco) supplemented with 10% FBS. DOK was cultured in DMEM added 10% FBS and 5 *μ*g/mL hydrocortisone (Sigma). HOK was cultured in Oral Keratinocyte Medium (Sciencell). All cells were cultured in humidified 5% CO_2_ at 37°C.

### 2.5. Plasmid Transfection

The pENTER-vector and pENTER-APOBEC3H were purchased from Vigene Biosciences. Both plasmids had been verified by DNA sequencing before use. The plasmids were transiently transfected into HSC3 and HSC6 cells using Lipofectamine 3000 reagent (Invitrogen) for 4-6 h. After 36 h incubation, the transfected cells were harvested for use.

### 2.6. RNA Extraction

For total RNA extraction, TRIzol reagent (Invitrogen) was used to lysis cells. Then, chloroform was added to the mixture. After centrifuging, the RNA was precipitated by isopropanol, washed by ethanol, and resolved into RNase-free H_2_O. NanoDrop One spectrophotometer (Thermo Scientific) was used to quantify and qualify the RNA. RNA was stored at -20°C for use.

### 2.7. Quantitative Real-Time RT-PCR

The quantification of gene expression was performed by quantitative real-time RT-PCR using the LightCycler 96 system (Roche) and ChamQ™ SYBR® qPCR Master Mix (Vazyme) as previously described. The following primers were used: human APOBEC3H: F: 5′-AAGGCCCTCTTGTGTTACCAG-3′, R: 5′-CACTGCGTTTCGTCCAGTC-3′; human CXCL10: F: 5′-GTGGCATTCAAGGAGTACCTC-3′, R: 5'-TGATGGCCTTCGATTCTGGATT-3′; and human GAPDH: F: 5′-GAGTCAACGGATTTGGTCGT-3′, R: 5′-TTGATTTTGGAGGGATCTCG-3′. GAPDH was considered as an endogenous control.

### 2.8. Statistical Analysis

Data between the two groups were compared using a two-tailed unpaired Student *t*-test or Wilcoxon rank-sum test depending on the normality of data distribution. The correlations and estimated statistical significances were calculated by Spearman correlation analysis. Data were presented as the mean ± SD. *p* < 0.05 was considered significant.

## 3. Results

### 3.1. The Expression Pattern of AID/APOBECs in HNSC

**Figure 1:**
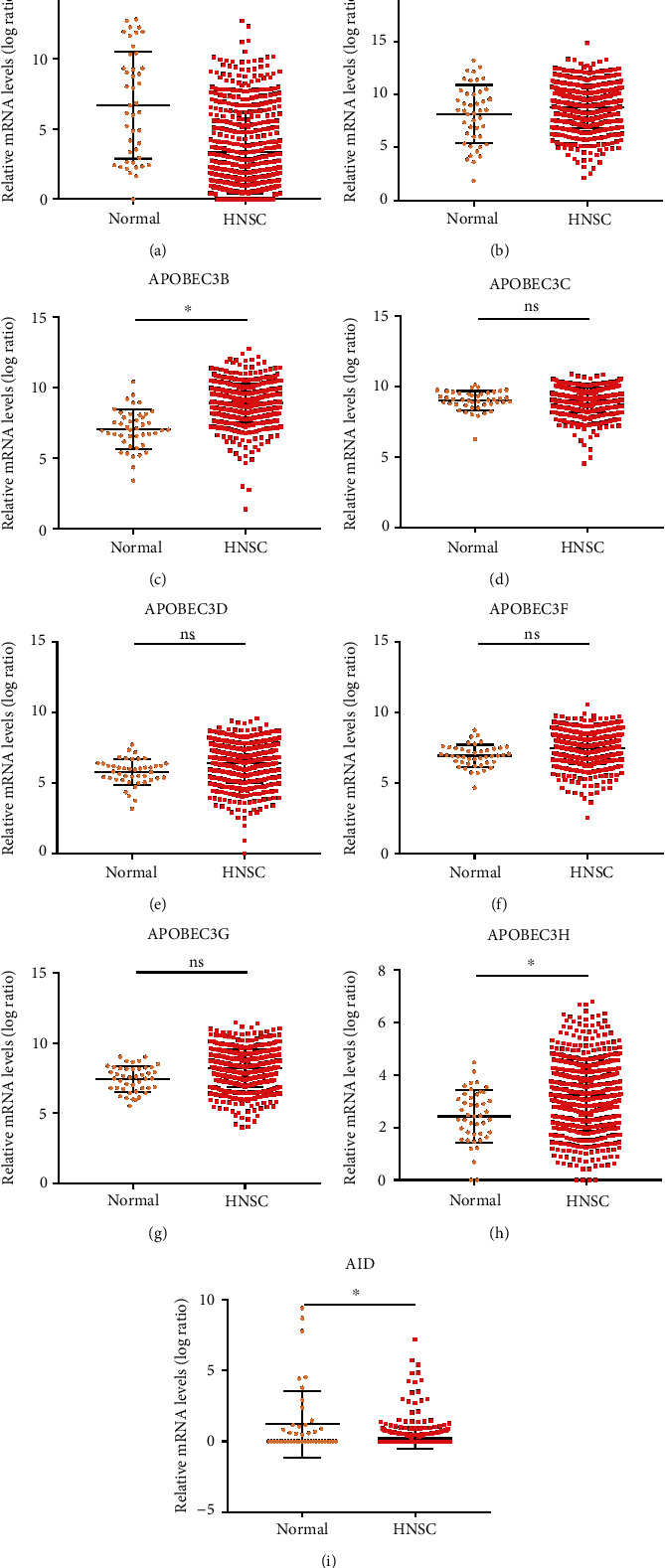
The AID/APOBEC expressions in HNSC. (a–i) The AID/APOBEC mRNA levels in HNSC primary tumor tissues (*n* = 530) and normal tissue control (*n* = 74). The values of mRNA expression are log2-based normalized count. Mean ± SD. ^∗^*p* < 0.05. ns: not significant. Student's *t*-tests.

The AID/APOBEC enzymes, including 9 family members (AICDA, APOBEC2, APOBEC3A, APOBEC3B, APOBEC3C, APOBEC3D, APOBEC3E, APOBEC3G, and APOBEC3H), were analyzed in this study. The mRNA expression levels of AID/APOBEC family members in the HNSC tumor tissues and normal epithelium tissues were calculated. The results showed that 2 out of 9 AID/APOBECs (APOBEC3A, APOBEC3B, APOBEC3D, APOBEC3G, and APOBEC3H) were upregulated, and 2 out of 9 AID/APOBECs (APOBEC2 and AID) were downregulated (Figure 1).

**Figure 2:**
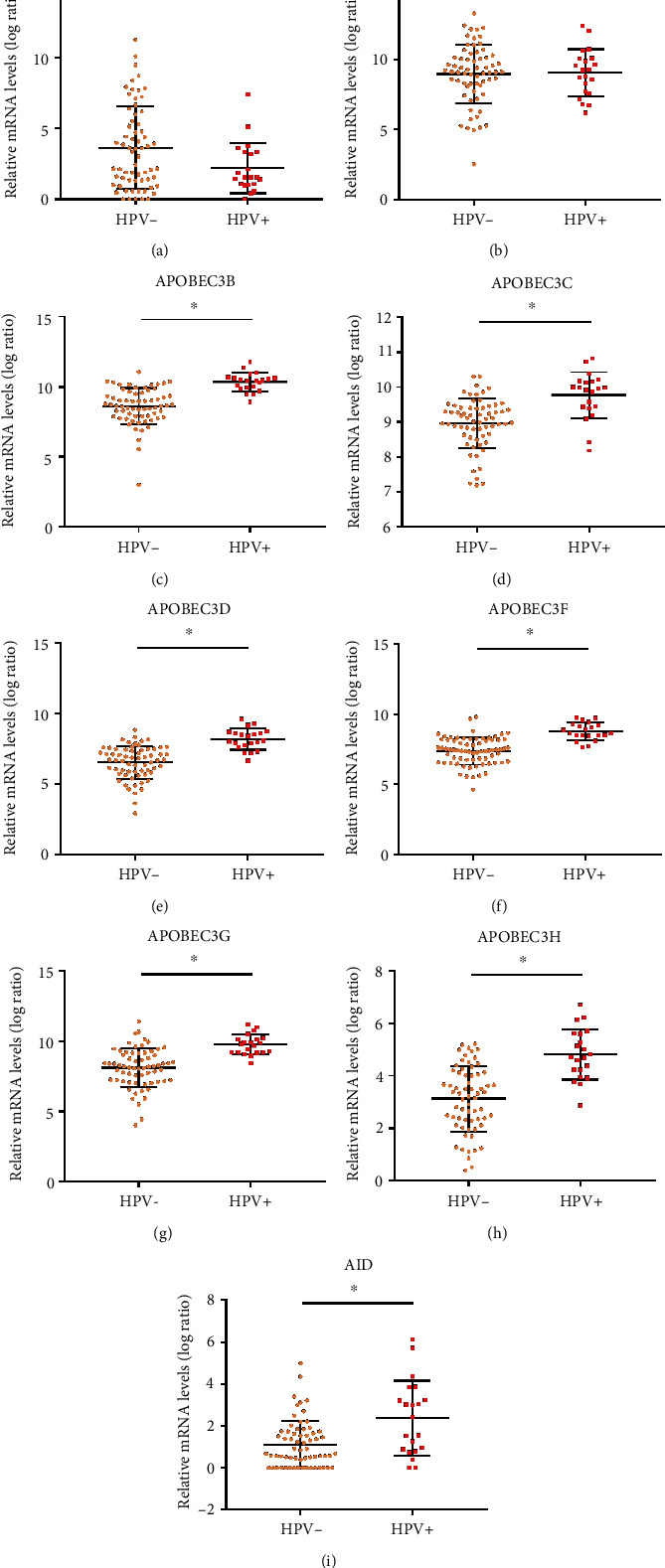
Correlations between AID/APOBEC expressions and HPV status in HNSC. (a–i) The AID/APOBEC mRNA levels in HPV+ (*n* = 22) and HPV- (*n* = 67) primary tumor tissues. The values of mRNA expression are log2-based normalized count. Mean ± SD. ^∗^*p* < 0.05. ns: not significant. Student's *t*-tests.

HPV+ HNSCs were reported to possess the highest burden of AID/APOBEC mutagenesis [31], leading us to hypothesize that HPV infection may be associated with AID/APOBEC abnormal expression patterns in HNSC. Hence, the expression levels of AID/APOBECs in different HPV infection statuses in HNSC were examined. We found that the expression levels of 7 out of the 9 (APOBEC3B, APOBEC3C, APOBEC3D, APOBEC3F, APOBEC3G, APOBEC3H, and AID) genes were significantly higher in the HPV-positive HNSC tissues than those in the HPV-negative HNSC tissues (Figure 2), which was similar with Henderson's findings [31]. Here, these results implied that HPV infection might be responsible for the abnormal expressions of AID/APOBEC enzymes in HNSC.

### 3.2. Relationships between AID/APOBEC Expression Patterns and Patients' Clinical Outcomes in HNSC

**Figure 3:**
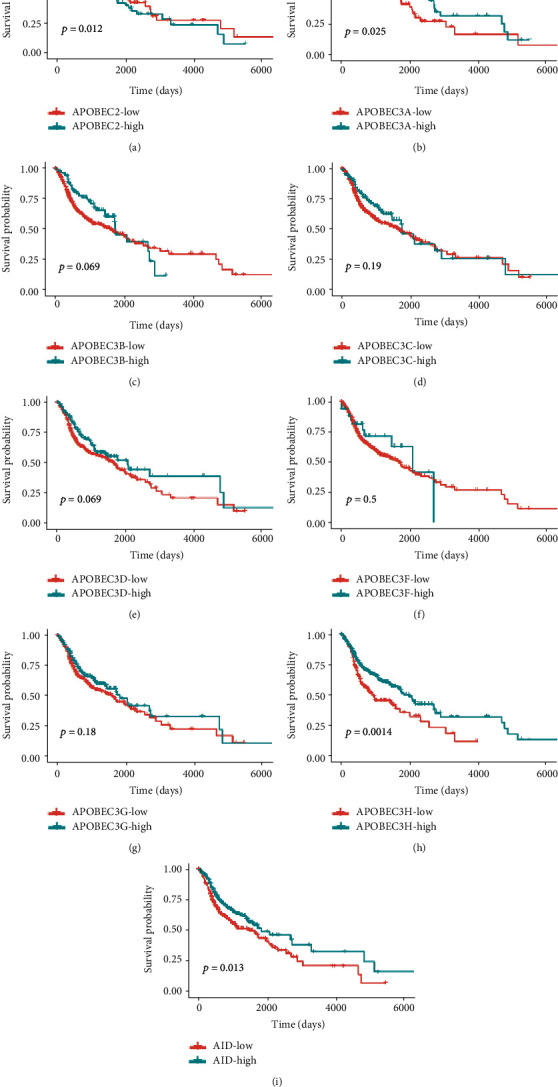
The prognostic values of AID/APOBEC mRNA levels in HNSC patients' overall survival. (a–i) Kaplan-Meier survival analysis was employed to determine the OS according to the mRNA levels of AID/APOBECs (high vs. low).

To explore the relationships between the AID/APOBEC mRNA levels and patients' clinical outcomes in HNSC, the Kaplan-Meier survival analysis was performed. The best cutoff values for the high and low groups of AID/APOBECs were selected according to the ROC analysis. The results revealed that APOBEC2, APOBEC3A, APOBEC3H, and AID showed predictive value in HNSC patients' overall survival (OS). Patients with high APOBEC3A, APOBEC3H, and AID mRNA levels had better OS than those with low mRNA levels (Figure 3). Interestingly, only APOBEC3H was upregulated and correlated with good clinical outcomes in HNSC, which attracted our attention.

Then, the univariate and multivariate cox regression analyses after correcting for age, alcohol, gender, HPV, stage, and smoking were performed to confirm the prognostic value of APOBEC3H in HNSC patients. APOBEC3H was confirmed to be an independent prognostic factor for HNSC patients (Supplementary Table 1). In addition, the prognostic values of APOBEC3H levels in HPV- and HPV+ HNSC patients, respectively, were also analyzed. The results revealed that HPV- patients with high APOBEC3H levels had better clinical outcomes than those with low APOBEC3H levels, while the two groups showed no survival differences in HPV+ patients (Supplementary Figure 1). Therefore, these data demonstrated that APOBEC3H might be a prognosis biomarker for HNSC patients.

### 3.3. Identification of APOBEC3H-Associated Methylation Pattern in HNSC

**Figure 4:**
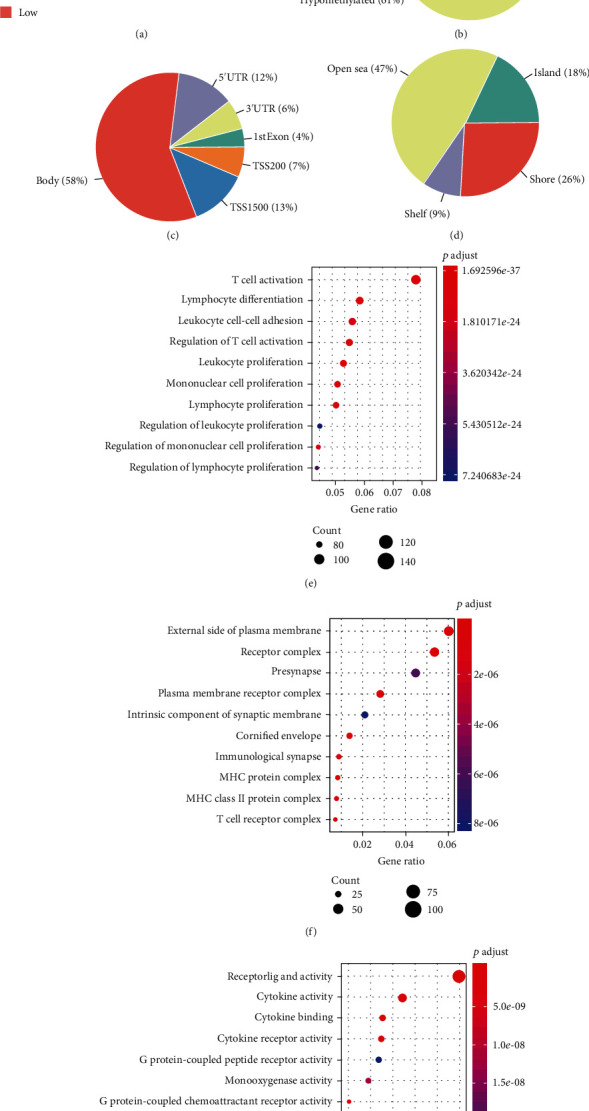
The genome-wide methylation pattern of APOBEC3H in HNSC. (a) The heatmap of APOBEC3H-associated methylation genes (columns, individual samples; rows, CpG sites; red, high methylation levels; blue, low methylation levels). (b) The pie chart of the ratios of hypomethylation and hypermethylation sites. (c) The proportion of differently methylated CpG site distribution according to the genome. (d) The proportion of differently methylated CpG site distribution according to the CpG island. Biological process (e), cell composition (f), and molecular function of the GO annotations (g) of APOBEC3H-associated methylation genes.

To explore the role of APOBEC3H in the genome-wide methylation regulation in HNSC, we compared the differently methylated genes in the high APOBEC3H expression group to the low expression group. The heatmap of APOBEC3H-associated CG sites is shown in Figure 4(a). Differential methylation analysis identified 1537 APOBEC3H-associated CG sites in HNSC samples, of which 934 (61%) were hypomethylated and 603 (39%) sites were hypermethylated (Figure 4(b)). Then, we explored the location-wise distributions of APOBEC3H-associated CG sites relative to the genomes and CpG islands. We identified that 32% APOBEC3H-associated CpG sites were located in the promoter regions, including 7% in TSS200, 13% in TSS1500, and 12% in 5′-UTR. Furthermore, 53% APOBEC3H-associated CpG sites were found to be located in or near the CpG islands, including 18% in the CpG islands, 26% in the shore, and 9% in the shelf of the CpG islands (Figures 4(c) and 4(d)).

**Figure 5:**
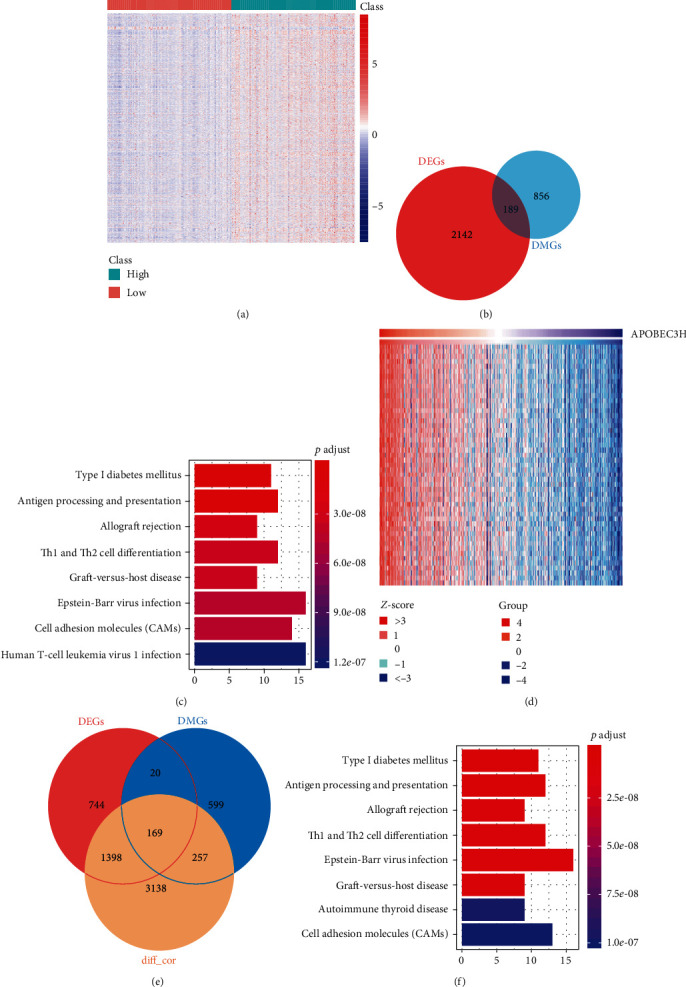
Biological functions of APOBEC3H in HNSC. (a) The heatmap of APOBEC3H-specific mRNAs (columns, individual samples; rows, genes; red, high expression levels; blue, low expression levels). (b) Venn diagram of the intersecting DEGs and DMGs from APOBEC3H-specific methylation genes (red, DEGs; blue, DMGs; cross area, both DEGs and DMGs). (c) The KEGG analysis of the common 189 genes in both gene clusters. (d) The heatmap of APOBEC3H-coexpressed genes identified by TIMER (*Z*-score, expression levels of each individuals; Group, expression levels of APOBEC3H). (e) Venn diagram of the intersecting genes from DMGs, DEGs, and APOBEC3H-coexpressed genes (diff_cor). (f) The KEGG analysis of the common 169 genes in above three gene clusters.

Next, the biological process (BP), cell composition (CC), and molecular function (MF) of GO enrichment analysis were employed to explore the underlying roles of differentially methylated genes (DMGs). Terms were arranged in ascending order according to the adjusted *p* values and listed the top 10 ones. The results showed that the DMGs were enriched in biological processes, such as T cell activation and lymphocyte differentiation (Figure 4(e)); in cell composition, such as external side of plasma membrane and receptor complex (Figure 4(f)); and in molecular functions, such as receptor ligand activity and cytokine activity (Figure 4(g)). Considering that APOBEC3H preferred to deaminate TTC sequence [19], the different methylation levels of TCG between the APOBEC3H-high and APOBEC3H-low groups were also identified. The results were similar with the findings in Figure 5 (Supplementary Figure 2). Therefore, those data indicated that the deamination-dependent demethylation activity of APOBEC3H might play critical roles in regulating HNSC immune responses.

### 3.4. Biological Functions of APOBEC3H in HNSC

To explore the correlations between the methylation levels of APOBEC3H-mediated DMGs and the gene expression levels, we also analyzed the differently expressed genes (DEGs) between the APOBEC3H-high expression group and low expression group. A total of 2331 DEGs were identified, including 1942 upregulated and 389 downregulated mRNAs. 189 genes were identified to be differently methylated and expressed in the APOBEC3H-high group (Figures 5(a) and 5(b)). The KEGG analysis showed that these 189 genes were mainly enriched in antigen processing and presentation, and Th1 and Th2 cell differentiations (Figure 5(c)).

Next, we examined genes whose expression levels were correlated with APOBEC3H expression levels using TIMER. As the results showed that a total of 4812 genes were confirmed to be positively related to APOBEC3H. Among which, 169 genes were hypomethylated, upregulated, and positively correlated with APOBEC3H expression (Figures 5(d) and 5(e)). The KEGG analysis also implied that these genes were mainly concentrated in antigen processing and presentation, and Th1 and Th2 cell differentiations (Figure 5(f)). Hence, these findings demonstrated that APOBEC3H might regulate the immune activity through its deamination-dependent demethylation activity in HNSC.

### 3.5. The Relationship among APOBEC3H, CXCL10, and CD8+ T Cell Infiltration

**Figure 6:**
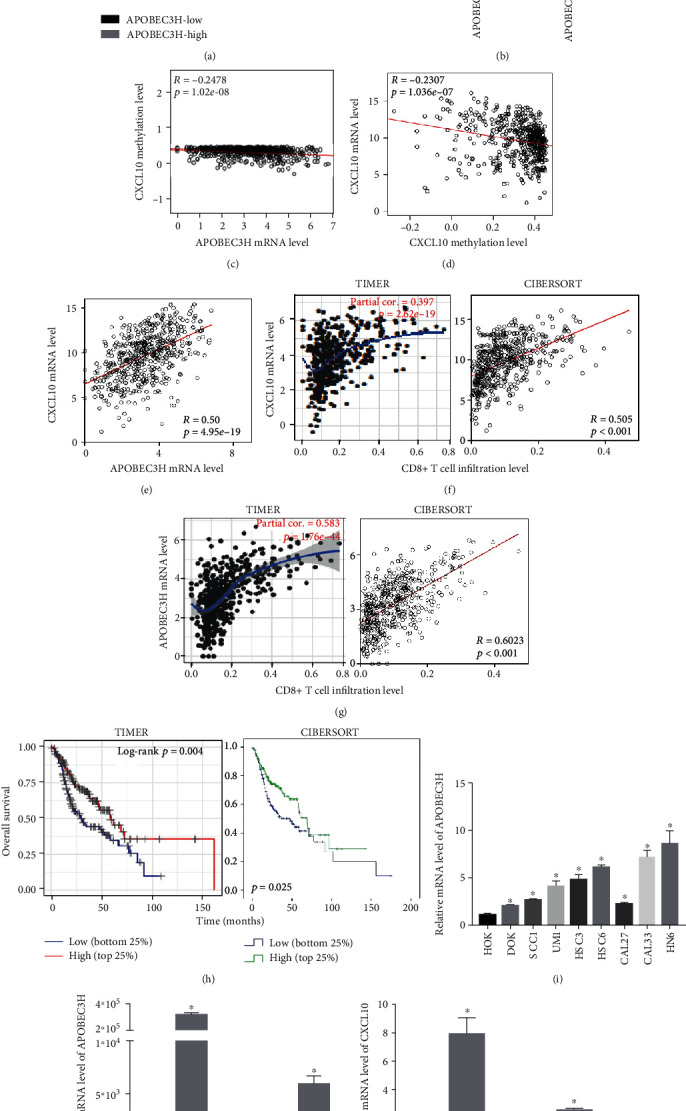
Correlations among APOBEC3H, CXCL10, and CD8+ T cell infiltration in HNSC. (a) The differentially methylated CpG sites of CXCL10 in the APOBEC3H-high and APOBEC3H-low groups. (b) The mRNA levels of CXCL10 in the APOBEC3H-high and APOBEC3H-low groups. (c–g) Spearman analysis of the correlations between CXCL10 methylation levels and APOBEC3H mRNA levels (c), CXCL10 mRNA levels and methylation levels (d), APOBEC3H and CXCL10 mRNA levels (e), CXCL10 mRNA levels and CD8+ T cell infiltration (f), and APOBEC3H mRNA levels and CD8+ T cell infiltration (g) in HNSC tumors. Data were analyzed using the TIMER and the CIBERSORT dataset. (h) Kaplan-Meier analysis was employed to determine the OS according to the CD8+ T cell infiltration (high vs. low). Data were analyzed using the TIMER dataset. (i) The qPCR results of APOBEC3H mRNA levels in normal cell line (HOK), dysplasia cell line (DOK), and HNSC cell lines (SCC1, UM1, HSC3, HSC6, CAL27, CAL33, and HN6). (j, k) The overexpression plasmids (APOBEC3H and Vector) were transiently transfected in HSC3 and HSC6 cells. The qPCR analysis was performed to confirm (j) the transfection efficiencies of APOBEC3H and (k) the mRNA levels of CXCL10. Mean ± SD. ^∗^*p* < 0.05. Student's *t*-tests.

CXCL10, which plays essential roles in helping CD8+ T cell trafficking and infiltration into the tumor microenvironment [32], was substantially hypomethylated in the promoter region and upregulated in the APOBEC3H-high group (Figures 6(a) and 6(b)). The promoter methylation level of CXCL10 was negatively correlated with APOBEC3H mRNA levels, and the mRNA level of CXCL10 was positively correlated with APOBEC3H mRNA levels (Figures 6(c) and 6(d)). Furthermore, CXCL10 and APOBEC3H expression levels were both positively correlated with CD8+ T cell infiltration in HNSC tumors (Figures 6(e) and 6(f)). Higher CD8+ T cell infiltration exhibited better prognosis in HNSC patients (Figure 6(g)).

To further validate those findings, we assessed the expression levels of APOBEC3H in HNSC cell lines. The qPCR results showed that APOBEC3H was upregulated in all HNSC cell lines and dysplasia cell line DOK compared to the normal epithelial cell line HOK (Figure 6(h)). Transiently transferring APOBEC3H plasmid to HNSC cell lines (HSC3 and HSC6) could obviously upregulate CXCL10 mRNA levels in contrast to transferring vector plasmid (Figures 6(i) and 6(j)). Collectively, those data implied that APOBEC3H might regulate the immune activity through upregulating CXCL10 in HNSC.

## 4. Discussion

Given the encouraging results archived in the clinical trials of anticancer immunotherapy, HNSC represents one of the most promising malignancies of immunotherapy research due to its immunosuppressive character [33]. However, the low response rate of the current immunotherapy strategy limits its application in HNSC. Identifying the underlying mechanisms of immune response activity may provide a novel insight into HNSC immunotherapy. In consideration of the essential roles of AID/APOBEC cytidine deaminase in cancer evolution and immune activation, we focused our insights on the AID/APOBECs in HNSC. Here, we comprehensively analyzed the roles of AID/APOBECs in HNSC and found that APOBEC3H was substantially upregulated in HNSC. HPV+ tumors showed higher APOBEC3H expression than HPV- ones. Patients with high APOBEC3H levels had better overall survival. In comparison with the methylation pattern with low APOBEC3H expression tumors, tumors with high APOBEC3H levels exhibited genome-wide DNA hypomethylation. Furthermore, APOBEC3H was found to be negatively associated with CXCL10 methylation and positively associated with CXCL10 expression. Both APOBEC3H and CXCL10 expressions were linked to CD8+ T cell infiltration in tumors. Our cell-based experiments confirmed that APOBEC3H could upregulate CXCL10 expression *in vitro*. Taken together, APOBEC3H might increase CD8+ T cell infiltration into HNSC tissues through CXCL10 demethylation and upregulation, which might provide a potential biomarker for HNSC immunotherapy.

APOBEC mutations, especially APOBEC3 mutations, have been reported to be tightly linked to immune activation and infiltration via induction of IFN-*γ* in HNSC [9]. In addition to the mutagenic roles, several AID/APOBECs could also play critical roles in DNA demethylation [17, 34]. The AID-mediated DNA demethylation was the most intensely studied among all the family members [35–37]. AID could initiate mismatch repair (MMR) and base excision repair (BER) by deamination of 5-methylcytosine, causing dmC to be replaced by dC, and eventually demethylated the target genes [38], which was considered to be required for stem cell pluripotency reprogramming and mouse primordial germ cell development. In addition, APOBEC1 and APOBEC2 were also postulated to implicate in DNA demethylation [39, 40]. The demethylation activity on oncogenes or tumor suppressor genes mediated by AID/APOBEC was thought to affect tumor occurrence, development, and metastasis. However, the expression pattern and deamination-dependent demethylation roles of AID/APOBEC cytidine deaminases in HNSC remain unknown. Here, through a comprehensively analysis of the AID/APOBEC expression patterns and clinical characteristics, we identified that APOBEC3H was aberrantly upregulated. Surprisingly, higher APOBEC3H expression was associated with good clinical outcomes in HNSC. APOBEC3H is the most polymorphic one of seven APOBEC3 family members, which has been identified seven haplotypes (hap I–VII) and four mRNA splicing variants and demonstrated playing a potential function of genomic mC modification [41]. Thus, we focused our insight on the demethylation activity of APOBEC3H in this study. In tumors with high APOBEC3H expression levels, a genome-wide DNA hypomethylation pattern was observed. Notably, the APOBEC3H-associated methylated genes were enriched in immune activity, such as antigen processing and presentation, and T cell differentiation and activation, implying that APOBEC3H might regulate the immune response via its demethylation activity in HNSC.

Chimeric antigen receptor (CAR) T cell immunotherapy, which is genetically engineered T cells to express a certain receptor that recognizes a specific antigen, has given rise to breakthrough in anticancer clinical trials, like hematological malignancies [42]. Given that CAR-T cells are difficult to traffic into solid tumors, more efforts are need to be made in increasing CAR-T treatment effects in solid tumors [43]. Recently, CAR-T cells were used to treat HNSC in a phase I clinical trial (NCT01818323). The EGFR-target CAR-T cells targeted to hypopharyngeal squamous cell carcinoma have been reported to acquire an encouraging effect [44]. The CD70-specific CAR-T cells could specifically recognize and efficiently eliminate CD70-positive HNSC cells [45]. Given the critical roles of CXCL10 in regulating immune responses through activating and recruiting leukocytes such as T cells, eosinophils, monocytes, and NK cells, several groups indicated that increasing CXCL10 production of tumor cells might facilitate CAR-T cell treatment efficiency in solid tumors [46, 47]. In this manuscript, we revealed that HNSC patients with high APOBEC3H levels were linked to high CXCL10 expression and CD8+ T cell infiltration into HNSC tissues. Therefore, we assumed that APOBEC3H expression could be a biomarker for predicting CD8+ CAR-T treatment efficiency in HNSC, and appending exogenous APOBEC3H might be a potential strategy for HNSC immunotherapy.

HPV infection has a rapidly increasing incidence and is established to be a cause of HNSC tumorigenesis. The response to the current treatment of HPV+ HNSC is sharply different from that of HPV− HNSC [5]. HNSC patients with HPV+ are also associated with more favorable clinical outcomes than those with HPV-. More importantly, the prognostic role of HPV infection has been defined by the eighth version of the *American Joint Committee on Cancer* (AJCC). However, the exact reason for the role of HPV in improving patients' prognostication is less well known, and treatment decision based on this guideline used to select patients has not been recommended. Thus, to understand the mechanisms of HPV infection in improving HNSC patients' survival is a major interest of several research teams, including us. Recently, HPV infection-induced immune activation has been demonstrated to be responsible for its good effects on anticancer therapy [48]. Virus, including HPV infection, has been reported that it could upregulate AID/APOBEC gene expression. HPV infection normal breast epithelial cells could cause APOBEC3B mRNA upregulation along with *γ*-H2AX foci formation and DNA damage increase [48]. Henderson and colleagues also found that compared to HPV- cases in HNSC, HPV+ ones exhibited elevated APOBEC3B levels and displayed PIK3CA helical domain mutations [31]. HPV infection in HNSC and cervical cancers increased the expression level of APOBEC3B through the viral oncoprotein E6 [49]. However, the effects of HPV infection on other AID/APOBECH enzyme expressions remain uncertain. In this study, we performed a comprehensive analysis of the relationships between AID/APOBEC expression and HPV infection status, and found that eight out of eleven AID/APOBEC genes were upregulated in HPV+ HNSC tumors. Considering the potential role of APOBEC3H in improving CD8+ T cell infiltration, we partially provide an explanation for the good prognosis of HPV+ HNSC patients based on their high APOBEC3H expression.

To our knowledge, this is the first study on the comprehensive analysis of APOBE3H in HNSC. APOBEC3H was identified to be a potential prognostic predictor and therapeutic target for oncoimmunotherapy in HNSC, in consideration of its essential role in regulating CXCL10-mediated immune activation and CD8+ T cell infiltration into the tumor microenvironment. Our findings broadened the knowledge that AID/APOBECs might regulate the immune activity not only through its mutagenic activity but also through its demethylation activity in HNSC.

## Data Availability

The data used to support the findings of this study are included in this paper.

## Supplementary Materials

Supplementary Materials Table 1: relationship between APOBEC3H and overall survival in HNSC. Figure 1: the prognostic value of APOBEC3H expression levels in HPV- and HPV+ HNSC patients' overall survival. Figure 2: the genome-wide methylation pattern for TCG enrichment of APOBEC3H in HNSC.
