# Supplementary material for: Association between APOBEC3H-Mediated Demethylation and Immune Landscape in Head and Neck Squamous Carcinoma
Source: Biomed Res Int. 2020 Jul 24;2020:4612375. doi: 10.1155/2020/4612375 (PMC7397441; doi:10.1155/2020/4612375)
Supplement: Supplementary Materials — Table 1: relationship between APOBEC3H and overall survival in HNSC. Figure 1: the prognostic value of APOBEC3H expression levels in HPV- and HPV+ HNSC patients' overall survival. Figure 2: the genome-wide methylation pattern for TCG enrichment of APOBEC3H in HNSC. [file 4612375.f1.docx]

**Supplementary materials**

**Table 1: Relationship between APOBEC3H and overall survival in HNSC**

|  | HR | 95% CI | *p* value |
| --- | --- | --- | --- |
| Crude model | 0.59 | 0.45-0.79 | 0.0003 |
| Model I | 0.59 | 0.44-0.79 | 0.0003 |

Crude model: we did not adjust other covariants

Model I adjust for age, alcohol, gender, HPV, stage, smoking

HR: hazard ratio; CI: confidence interval


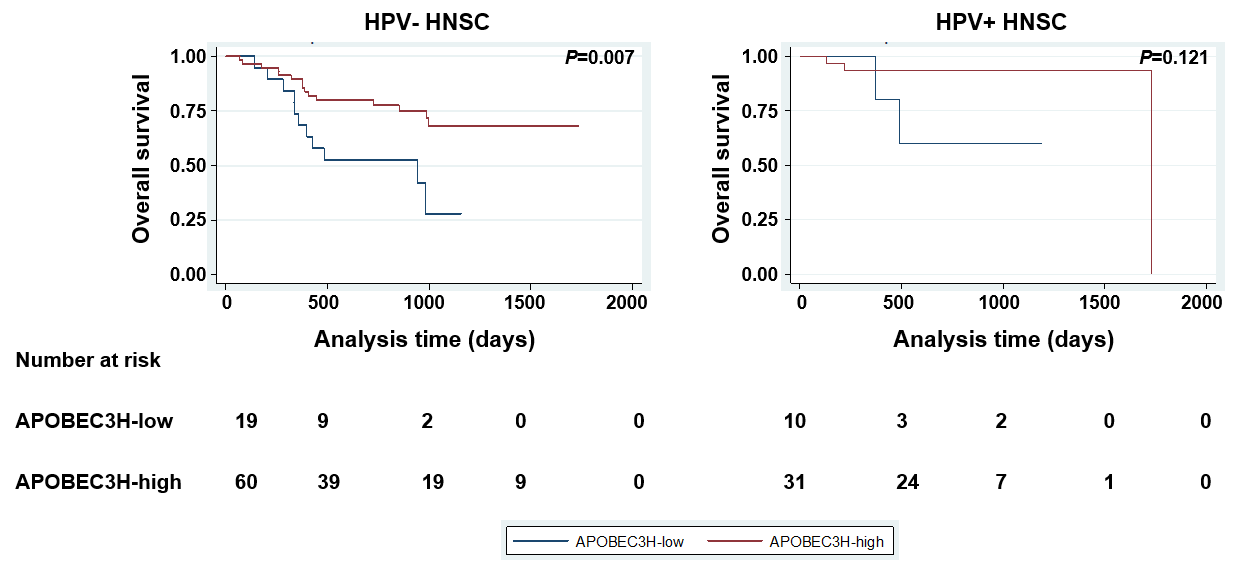


**Figure 1. The prognostic value of APOBEC3H expression levels in HPV- and HPV+ HNSC patients’ overall survival.**


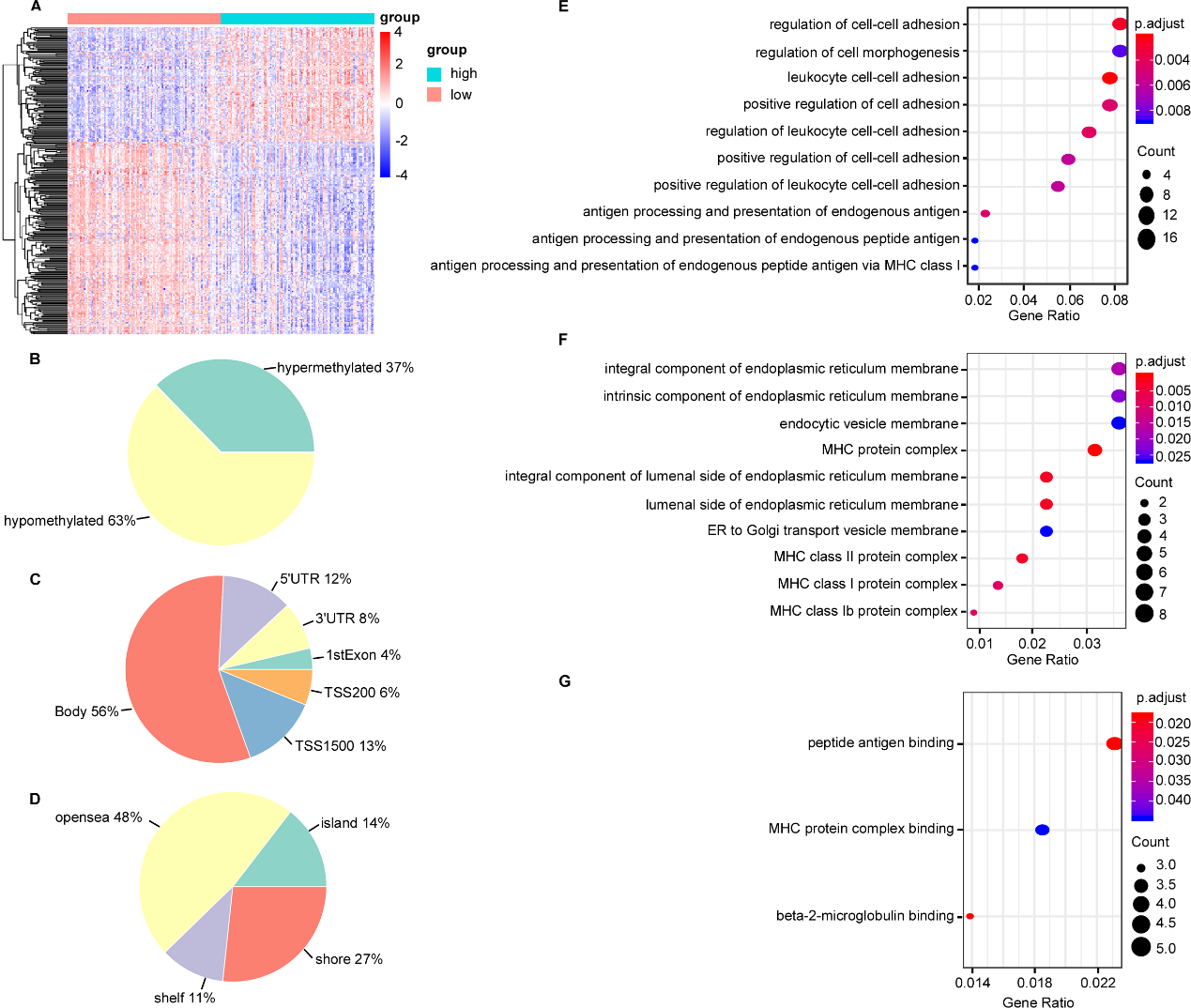


**Figure 2.** **The genome-wide methylation pattern for TCG enrichment of APOBEC3H in HNSC.** (A) The heatmap of APOBEC3H-associated TCG site methylation genes. Columns, individual samples; rows, CpG sites. Red, high methylation levels; blue, low methylation levels. (B) The pie chart of the ratios of hypomethylation and hypermethylation TCG sites. (C) The proportion of aberrantly TCG methylated CpG sites distribution according to the genome. (D) The proportion of aberrantly TCG methylated CpG sites distribution according to the CpG island. (E-G) (E)Biological process, (F) cell composition and (G) molecular function of the GO annotations of APOBEC3H-associated TCG site methylation genes.
